# HIV Risk among MSM in Senegal: A Qualitative Rapid Assessment of the Impact of Enforcing Laws That Criminalize Same Sex Practices

**DOI:** 10.1371/journal.pone.0028760

**Published:** 2011-12-14

**Authors:** Tonia Poteat, Daouda Diouf, Fatou Maria Drame, Marieme Ndaw, Cheikh Traore, Mandeep Dhaliwal, Chris Beyrer, Stefan Baral

**Affiliations:** 1 Department of International Health, Johns Hopkins School of Public Health, Baltimore, Maryland, United States of America; 2 Enda Santé, Dakar, Senegal; 3 Gaston Berger University, St-Louis, Senegal; 4 United Nations Development Program, New York, New York, United States of America; 5 Department of Epidemiology, Johns Hopkins School of Public Health, Baltimore, Maryland, United States of America; 6 Center for Public Health and Human Rights, Johns Hopkins School of Public Health, Baltimore, Maryland, United States of America; Tulane University, United States of America

## Abstract

Men who have sex with men (MSM) are at high risk for HIV in Senegal, with a prevalence of 21.5%. In December 2008, nine male HIV prevention workers were imprisoned for “acts against nature” prohibited by Senegalese law. This qualitative study assessed the impact of these arrests on HIV prevention efforts. A purposive sample of MSM in six regions of Senegal was recruited by network referral. 26 in-depth interviews (IDIs) and 6 focus group discussions (FGDs) were conducted in July–August 2009. 14 key informants were also interviewed. All participants reported pervasive fear and hiding among MSM as a result of the December 2008 arrests and publicity. Service providers suspended HIV prevention work with MSM out of fear for their own safety. Those who continued to provide services noticed a sharp decline in MSM participation. An effective response to the HIV epidemic in Senegal should include active work to decrease enforcement of this law.

## Introduction

Senegal is often considered to be a success story with respect to HIV prevention among sub-Saharan African countries [1]. With an estimated adult HIV prevalence of approximately 1%, it has one of the lowest rates in the region [2]. However, Senegal has a burgeoning epidemic among MSM, with most recent estimates of HIV prevalence in this population approaching 22% [3].

In the early 1990s, a landmark ethnographic case study of male homosexuality was conducted in Senegal [4]. This study was crucial in making the case for the existence of same-sex sexuality in Senegal and the need to address the sexual health of this hidden population. In 2001, a mixed-methods study of HIV risk among MSM in Senegal was conducted [5], [6]. In addition to information on sexual identity and practices, this study provided important insights about the role of violence and stigma in the lives of MSM and the dearth of appropriate and accessible targeted health services. In response, Senegalese MSM partnered with non-governmental organizations (NGOs) to implement HIV prevention programs in many parts of the country [7], [8]. Moreover, the national Senegalese AIDS strategy identified MSM as a key target population for prevention, and the Senegalese Ministry of Health implemented innovative programs to reach and serve these men [9].

One of the earliest studies of HIV prevalence among MSM in Africa was completed in Senegal in 2004 [10]. The sample of 463 MSM, recruited via snowball sampling from five urban communities, demonstrated an HIV prevalence of 21.5% (95% CI 17.8–25.6), and many men in this study reported high risk sexual practices. A follow-up study of 501 MSM in Senegal completed three years later confirmed an HIV prevalence of 21.8% (95%CI: 18.3–25.7) [3] and found a significant increase in condom use during sex with both men and women. The authors concluded that prevention campaigns and HIV care and support programs conducted in Senegal among MSM were followed by a reduction of risk-taking practices among this population [3].

To date, despite early calls for more ethnographic research on African same-sex practices [4] and a growing body of epidemiologic literature on the burden of HIV among MSM in Africa [11]–[14], limited qualitative research has been published on this topic [4]–[6], [15]–[17]. Understanding the complex contexts in which MSM in Africa face such disproportionate risk for HIV is critical for the sustainability of effective interventions [17]. Individual level risks often result from contextual layers of risk that are beyond individual control.

Beyrer and Baral have described a modified multilevel ecological model of HIV risk [18]. At the core are individual-level risks such as condom use, number of sexual partners, and genital ulcerative diseases. At the level of social and sexual networks, factors such as multiple and concurrent partnerships increase risk. The community level includes stigma, sexual norms, and access to antiretroviral medications. At national and political levels, human rights contexts impact all other levels. In particular, Beyrer describes criminalization and stigmatization of homosexuality as drivers of HIV risk among MSM in developing countries.

Social capital is a concept that cuts across multiple levels of the modified ecological model. The World Bank defines social capital as “institutions, relationships, attitudes, and values that govern interactions among people and contribute to economic and social development [19].” Two main elements of social capital have been described in the literature: structural social capital, such as community structures, networks, institutions, and rule of law in a state; and cognitive social capital including social norms, shared values, and mutual trust [19], [20]. Thus, in the modified ecological model, cognitive social capital operates at the level of the individual and network, while structural social capital operates at the level of the network, community, and nation. The importance of social capital has been increasingly recognized as a major social determinant of health due to its association with health outcomes, including chronic disease-related morbidity and mortality and more recently sexually transmitted infections [13], [21]–[30]. Moreover, several studies have suggested that the implementation of interventions that address social capital among MSM can potentially decrease marginalization, stigma, and the risk for HIV infection [21], [22], [31]–[34].

State-sponsored crackdowns against homosexuality in countries such as Senegal, Malawi, Kenya, Uganda, and Zimbabwe have received widespread international press over the last few years [35]–[38]. Same-sex practices are criminalized in 38 of the 53 African nations [8]. In Senegal specifically, Article 319 of the Penal Code, n° 66-16 dated 1 February 1966, translates as follows:

*…whoever will have committed an improper or unnatural act with a person of the same sex will be punished by imprisonment of between one and five years and by a fine of 100,000 to 1,500,000 CFA* [39].

In December 2008, the 15th International Conference on AIDS and STIs in Africa (ICASA) was held in Dakar. Despite the existing penal code, Senegalese public health officials publicly pledged their support for reducing HIV among sexual minorities. However, within weeks of the conference, police arrested nine male HIV prevention workers in Dakar for suspicion of engaging in homosexual conduct prohibited by national law [40]. In January 2009, the nine men were sentenced to eight years in prison and a fine of 500,000 CFA (approximately 1000 USD). They were given the maximum penalty for unnatural acts as well as an additional three years for conspiracy. The men remained imprisoned until Dakar's court of appeals subsequently overturned their conviction on April 20, 2009 [41]. The national and international media coverage of these events as well as other punitive actions against perceived homosexuals was extensive and has continued since the nine men were released [42].

Qualitative research is well-suited for the task of exploring the specific contexts within which people act and the influence of those contexts on their actions [43]. Theoretically grounded in the ecological model of HIV risk [18], this study used qualitative methods to examine how criminalization of homosexuality impacts HIV risk for MSM. This study sought to explore the impact of these highly publicized events on HIV prevention and treatment efforts among MSM in Senegal in the context of social attitudes toward homosexuality and the experiences of the arrested men who were embedded in social networks of MSM that span the country. Understanding the impact of the active enforcement of laws criminalizing same sex practices on the health of MSM is particularly critical to efforts to increase access to HIV prevention services and thus to reduce HIV risk for this vulnerable population in Senegal.

## Methods

### Ethics Statement

Ethical approval for this study was received from the Institutional Review Board at Johns Hopkins School of Public Health as well as the Conseil National de Recherche en Santé (CNRS) in Senegal. To maximize the safety of participants, no written materials describing the study were distributed, nor were written consent forms used. Prior to enrollment into the study, all recruited individuals provided verbal consent for participation.

During the approval process for this study, the ethical review board in Senegal (CNRS) was explicit about the need to maintain anonymity and confidentiality for all study participants. Several processes were put in place to do so, including the assignment of an anonymous code for each participant and the creation of a cover sheet for the verbal consent script that contained the participant code and basic demographic information. In order to further protect confidentiality, this cover sheet was kept separate from the transcript data. In addition, the CNRS explicitly approved the use of verbal consent and requested that study staff document receipt of verbal consent on the aforementioned cover sheet.

### Data Collection

A rapid appraisal approach was used for this study [44]. This approach allows for the production of qualitative results quickly in order to inform interventions. Rapid appraisal is characterized by three basic concepts that were reflected in the specific qualitative techniques chosen: (1) a system perspective was taken in the purposeful selection of participants, the use of focus group discussions (FGDs), as well as semi-structured in-depth interviews (IDIs) that included questions about social context; (2) triangulation of data collection involved the use of multiple research methods (ie. IDIs and FGDs), as well as the full participation of local researchers in the interdisciplinary team of data collectors and analysts; and (3) iterative data collection and analysis that took place with frequent meetings of the research team during data collection.

The study took place from July to August 2009. Data was collected in six geographically-distributed sites across Senegal including four urban sites (Dakar, Thiès, Mbour, and St. Louis) and two more rural settings (Kaolack and Ziguinchor). Participants included MSM and other key informants as described below.

#### MSM Participants

MSM participants were men drawn from MSM community-based organizations (CBOs) in the aforementioned six regions of Senegal. They were recruited using a non-probability, purposive sampling technique known as network referral. A network of collaborative MSM organizations in Senegal was used to identify potential participants who were then asked to recruit additional participants through messages spread by word-of-mouth. Inclusion criteria for MSM in this study were: born male, 18 years of age or older, and reporting ever having had oral or anal sex with another man.

The sample size for in-depth interviews with MSM included 26 men. Their ages ranged from 18 to 45 years, with an average age of 28 years. Twenty of them had never been married, and eleven had no children. Most men reported having both male and female sexual partners. Their occupations included students, tailors, restaurant workers, street vendors, business men, peer educators, and clinic mediators who provide linkages to care and who help other MSM navigate treatment services. Five participants admitted to paid sex work. All except two participants identified as Muslim and many were actively involved in religious association in their communities. Most of the men belonged to an association of MSM in their region and were recruited through the leader of that organization. Members from seven of the nine MSM CBOs in the country known to the authors participated. While some participants identified themselves as leaders of their local organization, they also clearly identified themselves as MSM and spoke directly of their own experiences and observations.

Interviews were conducted with 3 MSM in each of the six sites, except Mbour where 4 MSM were interviewed. In addition, 7 of the 9 men who had been arrested and imprisoned for acts against nature in December 2008 were interviewed. Six focus group discussions (FGDs) with MSM were held, one in each of the six regions. Each discussion included 6–9 individuals, for a total of 46 focus group participants.

Interviews were conducted in French or Wolof based on the choice of the participant. Each interview lasted between 25 and 120 minutes with an average duration of 60 minutes. The interviews elicited detailed narratives of individual experiences and perceptions. Specifically, each man was asked about his family and social life, sexual identity and practices, and health-seeking behavior before and after the arrests in December 2008. He was also asked to describe how he saw MSM represented in the media and the political and legal environment for MSM in Senegal.

All FGDs were conducted in Wolof and facilitated by experienced Wolof-speaking research staff. Focus group discussions (FGDs) with MSM provided information about community norms and variability. Participants were asked to discuss the family, social, legal, and political environment for MSM as a whole in Senegal, rather their individual experiences. Groups also discussed the impact of the December 2008 arrests on their local MSM association and on the sexual and health-seeking behavior of MSM in their region. The duration of FGDs ranged from 60 to 120 minutes. The average discussion lasted approximately 90 minutes.

#### Key Informants

The 14 key informants were drawn from health, legal, media, and academic institutions within the six regions described above. They were recruited by a Senegalese non-governmental organization (NGO) based in Dakar and included the following: 5 staff members from AIDS service organizations (ASOs) that provide services throughout the country; two attorneys who participated in the trial for the arrested men; one journalist from a popular national newspaper who authored articles on MSM during 2008 and early 2009; and one academic sociologist who specializes in research on homosexuality and who had spoken to the press on the issue of homosexuality in connection with the arrest. Each of these key informants was Senegalese and very engaged with issues of homosexuality, human rights, and public health during the arrests and the subsequent months.

Interviews were also conducted with one physician from each of the six regions, except St. Louis. That physician experienced an injury prior to the interview and was unable to participate. Physicians were purposively selected based on their clinical expertise in MSM health care needs, particularly HIV care and treatment.

Interviews with key informants were conducted in French and included questions about the political climate, challenges, and access to services for MSM. IDIs with journalists, academics, and attorneys focused on eliciting a deep understanding of the broad social systems and structural factors effecting MSM, while interviews with physicians and service providers sought to assess changes in both in the uptake of health care services and the ability to provide health care services for MSM since the arrests and subsequent conviction of the nine HIV prevention workers for acts against nature. None of the key informants openly identified as MSM.

### Analysis

To protect the confidentiality of the MSM study participants, all interviews and FGDs with MSM were transcribed and translated into French by the staff of the collaborating NGO. Interviews with key informants were directly transcribed into French by professional transcribers who were required to complete training in the ethics of human subjects research and to sign a confidentiality agreement.

Data for analysis included transcripts of audio recordings from the in-depth interviews and focus group discussions as well as expanded field notes from all data collection activities. A template organizing strategy, as described by Crabtree and Miller, was used for data analysis [45]. Using the computer software package Atlas.ti (version 6.0, Scientific Software Development GmbH, Eden Prairie, MN), *a priori* categorical codes based on the research questions were applied to the textual documents. Coded text was then extracted, organized by each categorical code, and read in multiple iterations by three different investigators in order to identify major themes related to contextual risks for HIV. Memos were used to organize the analytic process.

An important step in data validation included holding a community consultation with MSM study participants 6 months after the interviews were completed. Twenty-one MSM, including the seven men who had been arrested, participated in this meeting. During the meeting, preliminary findings from the study were presented and community members provided feedback on the interpretation of results as well as recommendations for ways forward. Due to the potential for identifiable information, especially for the seven men who had been incarcerated, permission to share and publish findings was explicitly and carefully sought from the MSM participants. At the conclusion of the meeting, the men confirmed the results presented below and gave permission for the results to be disseminated.

## Results

### Context for MSM in Senegal

#### Secrecy

Secrecy emerged as a major theme in response to stigma and discrimination among MSM in Senegal. Findings from both MSM and key informant interviews confirmed generally hostile societal attitudes toward MSM in Senegalese society. Participants described episodes of experienced or observed discrimination ranging from being refused a ride by a taxi driver to being violently attacked. When asked what would happen if a man were openly homosexual in public, the typical response was that he would be attacked and probably killed. Some MSM reported marrying women for the purpose of hiding or suppressing their attraction to men. In the face of such intense stigma, silence and secrecy were the norm. As an MSM participant in Ziguinchor stated:

*If my family knows my status as MSM, I think this will be the day I lose my parents, my family and my wife and I will not live long. The family will pray that I die because it is a shame.*

The need for secrecy sometimes extended to people who provided services for MSM. Several service providers reported the need to hide the fact that they provided services for MSM, even prior to the December 2008 arrests, because they were concerned about being stigmatized by family and friends.

The attitude of study participants toward criminalization of same sex practices was quite diverse. Some participants disagreed with the law and felt that it led to violence and arbitrary arrest of MSM and prevented them from fully living their lives. In addition, men who worked as clinic mediators and HIV educators found that their effectiveness was limited by this law because it required that they keep their work hidden. One participant stated:

*I am a health mediator. I am a father, an educator in the fight against HIV. I do not enjoy any protection from the law in relation to my safety in the exercise of my profession. To carry out our outreach, we are obliged to do so in secret.*

However, others felt that the law itself was not the problem, rather the way that it was applied and enforced. According to some participants, Penal Code Article 319 requires that people be caught *en flagrant délit* (in the act of sex); however, men have been arrested based on reports that they were homosexual without actually being caught having sex. Thus, if the law was correctly enforced, it would only be used against MSM who had sex in public or who were unsuccessful in hiding their sexual activity with men.

Many were concerned that efforts to change this law would lead to heightened attention and increased hostility and violence against MSM. Most felt that Senegal was not ready for such a change due to strong social and religious prohibitions against homosexuality. A few men expressed an internalized hatred for their own sexual desires and feared that decriminalization would mean that men would behave like women or have sex in the streets. Some felt the law was appropriate for a Muslim country and simply required MSM to keep their sexuality secret. A participant from Kaolack demonstrated this sense of internalized homophobia and the need for secrecy:

*The law is quite right because we are in a Muslim country. It is true that what is done is not good and therefore it is normal that the law condemns us… Senegal is an Islamic country, there are things you cannot do. And if you stay quiet at home, nothing can happen to you*.

Most men expressed the desire to have their sexual privacy respected rather than a desire to decriminalize same sex practices. The right to privacy was of primary importance to them. An MSM participant stated:

*I am not for legalization of homosexuality in a country that is 99% Muslim. What I advocate is respect for human rights. That MSM no longer be trapped or beaten in the streets or the victim of arbitrary arrest because of his sexual orientation… . To pass a law where gays have the freedom to do what they want as is the case in Europe is not my wish and that is unthinkable in Senegal… In my opinion, we just limit ourselves to advocate respect for human dignity where we are treated like human beings in our own right.*

### Sequelae of arrests on provision and uptake of HIV-related services

Three major themes emerged regarding the sequelae of the publicized arrests: (1) Negative publicity surrounding the arrests increased scrutiny and stigma of MSM. (2) Increased scrutiny heightened fear and increased hiding among MSM. (3) Provision and uptake of HIV prevention and treatment services decreased.

#### Negative publicity increased scrutiny and stigma

According to participants, most people were unaware that MSM existed in Senegal until the February 2008 publication in a local magazine of pictures depicting a marriage between two men in Senegal [46]. This led to significant media attention and MSM found themselves under increased scrutiny by the popular press and the public in general [35]. Publicity surrounding the arrests in December 2008 continued to keep the spotlight on MSM. An MSM participant in Dakar stated: “*Young people were afraid and are hiding because of the media who stir up the population against the MSM. If ever you're caught, you risk stoning or death.*” One of the men who had been arrested in December 2008 reported homophobia so widespread that he felt more comfortable in prison than on the street:

*The fact that you're an MSM and they are talking around your neighborhood. They are always talking on the radio, on TV about MSM and we must kill them. Even we who were in prison are more comfortable than those outside.*

Participants reported that press coverage of MSM was universally negative. Pejorative terms such as *goordjigen* (literally meaning man-woman in Wolof) and “*pédé*” (derogatory term for homosexual that can be translated as “queer” or “fag”) were commonly used in the popular press to refer to MSM. They felt that inflammatory language was used to sell newspapers. Newspaper articles frequently quoted religious leaders who called for expulsion (at best) and murder (at worst) for MSM in Senegal. During the ten month period between the publication of the article on marriage and the arrest of the nine HIV prevention activists in Dakar, more than 40 articles about MSM appeared in popular press, including those covering stories about men who were violently attacked by members of their communities and about a man whose dead body was twice exhumed and deposited on his family's front steps due to accusations of homosexuality made after his death. Participants who were active in HIV prevention reported resentment about this negative, one-sided portrayal of MSM. One participant in Dakar put it this way:

*Always the same language, they speak only on the negative side, never the positive. The MSM have played an important role in the fight against AIDS … but the media only talk about the negative side.*

This increased scrutiny and stigma even impacted the men who had been imprisoned. These men reported being singled out from other prisoners for mistreatment. They described being stripped naked, beaten, called insulting names, and prevented from showering because they were homosexual. As described by one former inmate:

*We have been tortured. I even think the word [torture] is too weak. I had done everything to hide my sexual orientation, and when I was arrested, the officer took the phone and called “Hello, the goordjigen are here.” Every day we had 10 blows with the baton in the morning and 10 blows in the evening. Other officers who were even from outside the police unit came to beat us. They insulted our mothers, fathers, and they treated us like goordjigen, pédes, etc. We were not entitled to the shower because we were homosexual.*

In addition to the physical and psychological mistreatment these men suffered while incarcerated, many described considerable social and mental health consequences of the arrest and subsequent disclosure of their sexual orientation to the public. They discussed being traumatized and considering suicide in the wake of this experience. Most of these men lost relationships with family and friends as well as their jobs and homes. When one man was asked how he handled the events during the arrest and after his release, he said:

*My release from prison has been very hard. I preferred even to kill myself. I was humiliated and insulted. In my neighborhood, my older sister was fighting because of the nasty things that were said against me. It was hard. After prison, it is fear I feel. I often think about death, but I never commit suicide… I think often of the day of my death.*

#### Increased fear and hiding

Participants reported an escalation of widespread fear and hiding among MSM as a result of the December 2008 events. They reported such fear of discrimination, violence, and arbitrary arrest that some MSM left their cities or even the country, which significantly affected sustainability of MSM CBOs. An MSM participant in Mbour noted:

*The consequences are many and varied, many men were afraid and this had an impact on operations [of the MSM CBO]. The president of our association has long been abroad, with the assistance of his partner, for fear of being denounced. Most young people wanted to leave the country. It was a very difficult time.*

After their release in April 2009, the men who had been incarcerated in December 2008 no longer felt safe providing prevention services and HIV treatment adherence support, thereby reducing access to services upon which their communities once relied. An MSM association leader in Dakar stated, “*For security reasons, we were obliged to stop our activities.*”

After the arrests, many men no longer felt safe attending meetings where they had previously received HIV education, condoms, and lubricant. They felt that people would recognize them as MSM and they would be subject to violence and detention. In addition to the loss of access to HIV prevention education and materials, this disruption in meetings affected the fledgling social networks among MSM communities. As one association member stated,

*MSM dare not receive or go to the talks or to seek condoms. They continue to have unprotected sex and that can bring other problems. Our association fell apart and half of our members are scattered in other regions.*

This fear and subsequent hiding also impacted MSM living with HIV being treated with antiretroviral therapy. A service provider in Dakar noted, *“This fear has had an impact at all levels. Some no longer even want to take their medicines or meet together for fear of being arrested.”* An MSM participant in Ziguinchor noted, *“The major impact of these arrests is fear. MSM are so afraid they do not want to leave their homes. They refuse to come to work and even go for treatment.”*

#### Decreased Provision of HIV Prevention Services

The nine men who were arrested in December 2008 were detained based on the presence of materials they used to provide safer sex education to their peers. One participant described what happened the night of the arrests and his efforts to explain their HIV prevention work to the police:

*When the police raided, they asked questions, searched the apartment. Although we justified the presence of certain tools such as counseling cards, condoms, dildos and all other activities we would do such as education and working with the Division of AID, this did not convince the police.*

Other MSM noted that prior to the events of December 2008, significant progress had been made in HIV prevention activities by MSM CBOs. Several participants noted that they were unaware of the need to practice safer sex with male partners until they attended sessions sponsored by community-based organizations. After learning about the risk of HIV and STI transmission at these workshops, they reported consistently using condoms with both male and female partners. Participants reported having access to condoms through the MSM CBOs and health care facilities. However, cost was cited as a barrier to use of condoms if they were not available from service providers.

While most of the men reported little difficulty getting condoms, many of them reported difficulty finding water-based lubricant. Even prior to the arrests in December, they often did not have money to buy appropriate water-based lubricants and resorted to the use of petroleum-based beauty products such as shea butter, scalp moisturizers, and body lotion for lubrication during anal intercourse. As a consequence, many men reported that condom breakage was a frequent occurrence. When MSM leaders went into hiding after the arrests in December 2008, MSM had increased difficulty accessing condoms and appropriate lubricants because they were unable to locate the people who had previously provided them.

Even organizations with a decade of experience providing HIV prevention services for MSM in Senegal lost enormous ground during the crisis surrounding the arrests. A service provider in Dakar stated:

*We had momentum then. We had established an MSM organization in each regional capital … . But when there were problems, the arrest of the MSM … the networks dissolved. People did no more activities, they did no more activities. They returned 10 years in reverse.*

The NGOs that had provided prevention services for these men actually suspended their HIV prevention work with MSM out of fear for their own safety. A service provider described her fear this way: “*I've seen programs on TV where religious leaders have called and threatened and I feared at one time to find [our facility] burned.*” Another organization reported not only suspending all MSM activities, but also taking down the sign for their organization in order to make it harder for the media to find them.

#### Decreased Uptake of HIV Prevention Services

Where prevention services were still available, many MSM were afraid to use them. An MSM participant in Dakar noted,

*The consequences are numerous and the MSM have returned underground. This does not mean they have stopped having sex. However, they do not care because they are afraid to pick up condoms.*

Service providers consistently reported increased difficulties in finding MSM, even when attempts were made to locate them. One service provider stated,

*Evidently, we've lost sight of many MSM despite our active attempts to find them; some have left town to go to the regions and among them were those who are sero-positive.*

Several of the service providers discussed the significant work that will be required to rebuild what has been lost in the wake of the crisis. Unlike their work 10 years ago when there was nothing, now they must overcome a new level of fear and loss of trust in organizations that were unable to prevent the crisis. One HIV prevention educator in Dakar put it this way:

*We have been involved in the fight against AIDS particularly on behalf of vulnerable groups. We organized sessions of talks, training on AIDS. We had gained positive results and had a grip on this target population up to the day when the events turned everything upside down. Many of the most committed players withdrew from the fight. A significant number of MSM have returned underground… Bisexuality exists among MSM. Even if taboo religiously and culturally, in terms of public health, it can have serious consequences for the spread of the epidemic. Now the MSM are convinced they are not protected despite their commitment.*

#### Decreased Uptake of HIV Care and Treatment Services

Prior to the events of December 2008, a national NGO had worked, in collaboration with the Senegalese Ministry of Health, with at least one medical provider in most regions to institute programs that facilitate care for MSM. These physicians had established trusting relationships with MSM in their communities and several MSM worked as clinic mediators to help educate their community about services available and to help them navigate the process of getting care from an MSM-supportive facility or provider. Some physicians waived consultation fees for MSM; and in some settings, HIV care and medications were free. These physicians had developed trusting relationships where MSM reported feeling comfortable disclosing their sexual orientation. Health care providers described the importance of knowing if the patient was MSM in order to provide appropriate care, treatment, and prevention counseling and services.

All of the physicians interviewed (representing 5 of the 6 regions studied: Thiès, Mbour, Kaolack, Ziguinchor, and Dakar) noticed a sharp decline in medical visits by MSM beginning in January 2009, after the arrests in December 2008, One doctor noted that many of his patients had left the country and discontinued their medical treatment. Several clinic mediators commented that men were afraid to seek HIV care for fear of violence. An MSM participant in Kaolack stated,

*If the hospital people know that you are MSM, you cannot go there for fear of being assaulted. I have a friend who was sick and was afraid to seek treatment from Dr [name withheld] after the events… he was afraid of being assaulted.*

A physician in Thiès anticipated the impact these events would have on his MSM patients with HIV. Several MSM participants who lived in the region reported that this physician called his patients and encouraged them continue their treatment and not to disrupt their care. Despite this active effort to retain HIV-infected MSM in care, some men were still too afraid to return to the hospital. In contrast to the fear expressed by MSM, all of the physicians interviewed denied any concerns for their own safety when providing services for MSM. All spoke of their continued commitment to serving the entire community, including MSM.

## Discussion

To our knowledge, this is the first study to examine the effects of increased enforcement of laws criminalizing same sex practices on HIV prevention and treatment efforts in the African context. Findings from this study suggest that the arrests of nine men for crimes against nature in Dakar in December 2008 led to reduced access to HIV prevention, care, and treatment for MSM well beyond the capital city. The increasing stigma and fear of violence associated with these arrests served as a disruption in the provision and uptake of these services.

Based on findings from this study, targeted stigma and discrimination against MSM resulted in human rights violations such as arbitrary arrests and violence. These abuses, in turn, lead to a decrease in provision and uptake of services both directly and indirectly. Directly, the nine men who were arrested were no longer able to provide services to their communities. Indirectly, the fear and hiding engendered by these abuses led to a decrease in uptake and provision of services, in part, through the loss of social capital.

Our findings suggest that the arrests in December 2008 reduced structural elements of social capital via direct and indirect pathways. The arrested men were embedded in social networks and community structures that spanned several regions of the country. Rumors as well as new reports about their experiences traveled from Dakar to as far away as Ziguinchor and influenced other MSM in their networks. When the MSM associations dissolved in the context of increased stigma, fear, and hiding, this disrupted these social networks and their relationships with health care institutions and preventive service organizations, thus potentially decreasing structural capital. In addition, social capital was lost at the network level when men stopped coming to association meetings and went into hiding due to fear. This study did not include a detailed assessment of the relationship between arrests and levels of cognitive social capital among MSM in Senegal. There is a limited evidence base and a lack of consensus on appropriate quantitative and qualitative tools to measure social capital among MSM and other sexual minorities in low and middle income country settings. Given the central role that social capital seemed to have played in this situation, it is crucial to both refine the tools and build the evidence base for characterizing the role of social capital in risk and resiliency as well as interventions for sexual minorities in low and middle income countries.

Many MSM reported significant benefits from HIV prevention services before the arrests of 2008, including learning of the need to use condoms with male partners. Moreover, they reported that this knowledge had translated into increased use of condoms with both male and female partners. The majority of condoms used were obtained through MSM CBOs or health facilities. This access to sexual health educational materials was impeded by the disruption in services, thereby increasing the likelihood of outcomes illustrated in the conceptual model: increased sexual risk practices (due to lack of access to condoms, lubricant, and information), decreased diagnosis and treatment of sexually transmitted infections (due to lack of uptake of treatment services), and ultimately increased risk of HIV acquisition and transmission.

In addition to the decreased uptake of HIV prevention and treatment services by MSM, this study found disruptions to or termination of the services provided by community based organizations, NGOs and health care workers secondary to these arrests. Service providers in this study reported difficulty in providing services out of fear that these services would be reported by the media and their organizations would suffer retribution. Well-established prevention programs serving hundreds of MSM stopped providing services after the arrests. These findings suggest that in stigmatizing environments where structural social capital is disrupted, even if there were funding for state-of-the-art comprehensive HIV prevention and care packages for MSM, organizations may choose not to provide these services in order to protect themselves. In the current economic environment in which donor funding appears to be increasingly limited, these findings have significant implications for effective use of existing resources.

Overall, our theoretical framework describes how social stigma, such as that described by MSM in Senegal, results in human rights violations that include government policies criminalizing same-sex practices, enhanced enforcement of laws criminalizing same sex practices, and targeted violence based on sexual discrimination. Furthermore, human rights violations create an environment where provision of preventive and clinical services is more difficult, resulting in lesser availability of services for MSM. Likewise, stigma, discrimination, and human rights violations impede the uptake of preventive and clinical care services for MSM. The participants also described significant mental health issues in response to the heightened stigma targeting MSM, manifesting in depression and even suicidal ideation. Studies have consistently demonstrated the link between mental health and higher risk sexual practices and decreased adherence to preventive and treatment programs, further highlighting the downstream effects of stigma [47], [48].

Disruptions in STI testing and treatment programs are expected to result in higher rates of undiagnosed or untreated genital and anal infections that can facilitate the transmission of HIV. Interruptions in HIV treatment give rise to selective mutations of HIV viruses that are harder to treat and lead to higher viral loads with higher potential for transmission. The downstream effects of stigma, discrimination, and human rights violations include lowering social capital and ultimately increasing unprotected anal sex and interrupting diagnostic and clinical care services – all of which are established risk factors for HIV acquisition and transmission among MSM.

The findings of this study corroborate the modified ecological model proposed by Beyrer and Baral [18] and provide explanatory mechanisms for how each level is associated with HIV risk. While individual level risk factors for HIV such as condom use are the most proximal to HIV risk, the prevalence of these risk factors is contexualized by higher order risk factors such as stigma and discrimination.

Some study participants noted that strict interpretation of the Penal Code Article 319, as written, does not ban same-sex practices but rather bans same-sex practices in public. However, men who interpret this law as requiring secrecy about their sexual behavior with other men may be hard to reach with prevention messages. The secrecy mandate impedes attendance at association meetings where HIV prevention education and safer sex materials could be obtained. In addition, some men in this study attempted to avoid accusations of homosexuality by marrying women and having children. This raises the concern that efforts to hide from the law may result in increasing HIV-related risk for both men and women [49].

The majority of the men interviewed for this study did not recommend working to repeal Penal Code Article 319 as an immediate policy and advocacy goal; instead they felt that this should be a longer term goal. Common themes that emerged from the key informant interviews and focus group discussions were that current strategic goals should be focused on changing interpretation or enforcement of this law. Specifically, participants felt that the positive right to privacy should supersede the enforcement of a law banning same sex practices. Despite the detrimental health effects of the law, as it is currently enforced, a wide variety of options could be collaboratively explored with key stakeholders to better serve the public health needs of both MSM and the general population.

A major limitation of this study is the recruitment of MSM participants from the immediate social networks of those most closely associated with the health organizations. While this purposive network sampling strategy draws strength from its ability to access a hidden population in multiple regions of the country, it also likely selected for participants who are more keenly aware of the relationship between health and human rights violations. Clearly, men who were socially connected enough to access prevention and care services prior to the arrests would have experienced the greatest impact from their disruption.

Given its limitations, the study allows for a deep appreciation of the health impact of criminalization and its enforcement on a network of MSM across several regions in Senegal. Using a collaborative research model that included civil society, the MSM community in Senegal, and academia, the study provides an example of a methodologically sound evaluation of the relationship between human rights violations and public health outcomes.

In conclusion, enforcement of criminal penalties on sex between men in Senegal limited the ability of health workers to provide essential HIV prevention services for MSM, including education, the provision of condoms and water-based lubricants, and treatment of sexually transmitted infections. Where services were available, active enforcement of the law reduced uptake of prevention and treatment programs among communities of MSM. This reduced uptake may have increased the risk of HIV among these men and their larger communities.

Responding to HIV among MSM calls for pragmatic public health approaches which are both evidence-based and rights-affirming. Punitive approaches increase vulnerability and limit these men's access to essential HIV and health services. The HIV, public health, and human rights communities need to be engaged in supporting evidence-based, comprehensive, and rights affirming approaches for MSM in Africa. This includes immediate efforts to decrease enforcement and longer term endeavors to repeal laws which criminalize same-sex practices.
